# Research Progress Regarding the Effect and Mechanism of Dietary Polyphenols in Liver Fibrosis

**DOI:** 10.3390/molecules29010127

**Published:** 2023-12-25

**Authors:** Jiayin Chang, Congying Huang, Siqi Li, Xiaolei Jiang, Hong Chang, Minhui Li

**Affiliations:** 1 Department of Pharmacy, Baotou Medical College, Baotou 014040, China; changjy567@163.com (J.C.); candysweethcy1209@163.com (C.H.); lisiwangyi2021@163.com (S.L.); jxl15147689596@163.com (X.J.); 2 Inner Mongolia Autonomous Region Hospital of Traditional Chinese Medicine, Hohhot 010020, China; 3 Inner Mongolia Key Laboratory of Characteristic Geoherbs Resources Protection and Utilization, Baotou 014040, China

**Keywords:** liver fibrosis, signaling pathways, dietary polyphenols, HSC, intestinal microbes

## Abstract

The development of liver fibrosis is a result of chronic liver injuries may progress to liver cirrhosis and liver cancer. In recent years, liver fibrosis has become a major global problem, and the incidence rate and mortality are increasing year by year. However, there are currently no approved treatments. Research on anti-liver-fibrosis drugs is a top priority. Dietary polyphenols, such as plant secondary metabolites, have remarkable abilities to reduce lipid metabolism, insulin resistance and inflammation, and are attracting more and more attention as potential drugs for the treatment of liver diseases. Gradually, dietary polyphenols are becoming the focus for providing an improvement in the treatment of liver fibrosis. The impact of dietary polyphenols on the composition of intestinal microbiota and the subsequent production of intestinal microbial metabolites has been observed to indirectly modulate signaling pathways in the liver, thereby exerting regulatory effects on liver disease. In conclusion, there is evidence that dietary polyphenols can be therapeutically useful in preventing and treating liver fibrosis, and we highlight new perspectives and key questions for future drug development.

## 1. Introduction

The global burdens of chronic liver disease, cirrhosis, and end-stage liver disease are increasing. Chronic liver disease is the eleventh leading cause of death and fourteenth leading cause of morbidity worldwide [1]. Liver fibrosis ranks eleventh in terms of mortality, and kills 100 million people annually [2]. Chronic liver injury is the principal manifestation of liver fibrosis [3] which is an abnormal wound repair reaction that is characterized by extracellular matrix (ECM) overdeposition and the abnormal hyperplasia of the connective tissue. In the absence of effective treatment, liver fibrosis can develop into cirrhosis or hepatocellular cancer [4]. Liver transplantation is currently the most effective treatment for cirrhosis; however, its clinical application is limited owing to the lack of donor material, limited expert technical support, and high associated costs [5]. Liver fibrosis has become a global epidemic affecting a wide range of people, and there is currently no specific and effective treatment [3,6]. Therefore, there is an urgent need to understand the underlying mechanisms and develop promising therapeutic strategies to treat liver fibrosis.

Hepatic stellate cells (HSCs) are a central driver of liver fibrosis in experimental and human liver injury, and they are the key cells responsible for ECM production [7,8,9]. After activation, HSCs transdifferentiate from vitamin A-storing cells to myofibroblasts, which are proliferative, contractile, inflammatory, and chemotactic cells that are characterized by their ability to facilitate ECM production following liver injury and in vitro [7,10,11]. HSCs have a distinct transcriptome profile that differentiates them from other types of resident liver cells. The cell-surface protein platelet-derived growth factor (PDGF) receptor-β, enzyme lecithin-retinol acyltransferase, the cytoskeletal proteins desmin and glial fibrillary acidic protein, and transcription factor heart- and neural crest derivatives-expressed protein 2 are among these markers [5]. The ECM is produced by myofibroblasts, smooth muscle cells, and pericytes, with myofibroblasts being the primary producers [12]. Fibrogenesis is expressed and there is an imbalance between the deposition and degradation of proteins in the ECM carried out by tissue inhibitors of metalloproteases (TIMPs) and degrading enzymes called matrix metalloproteinases (MMPs). This imbalance exacerbates the excessive accumulation of changes in the protein components of the ECM, including fibrillar collagen types I and III, α-smooth muscle actin (α-SMA), non-muscle myosin, fibronectin, and vimentin, which ultimately leads to the formation of scar tissue [13]. TIMP-1 and transforming growth factor (TGF-β) also promote anti-apoptotic signals and the survival of HSCs [14]. MMP-9 is capable of degrading collagen and gelatin in the ECM [15,16] and is associated with the breakdown of collagen and other matrix proteins in the cytoplasm of the activated HSCs. During liver damage, the continuous accumulation of ECM and an increase in collagen types I and III leads to the deposition of scars and liver fibrosis [17,18]. Oxidative stress is another pro-fibrogenic factor involved in the progression of liver fibrosis and is mainly attributed to the production of a large number of reactive oxygen species (ROS) and decreased antioxidant capacity [19,20]. Currently, there are three treatments for the regression of liver fibrosis by clearance of HSCs: apoptosis, senescence, and reversal [14]. During liver fibrosis, some signaling pathways and elements of these pathways are overactivated or inhibited, such as TGF-β/Smad and AMPK/mTOR. However, liver fibrosis is a dynamic process, and advanced fibrosis is reversible when pathogens are removed [21,22]. Additionally, gut microbes and their metabolites are thought to play significant roles in the development of liver fibrosis which should be considered when trying to reverse liver fibrosis.

**Figure 1:**
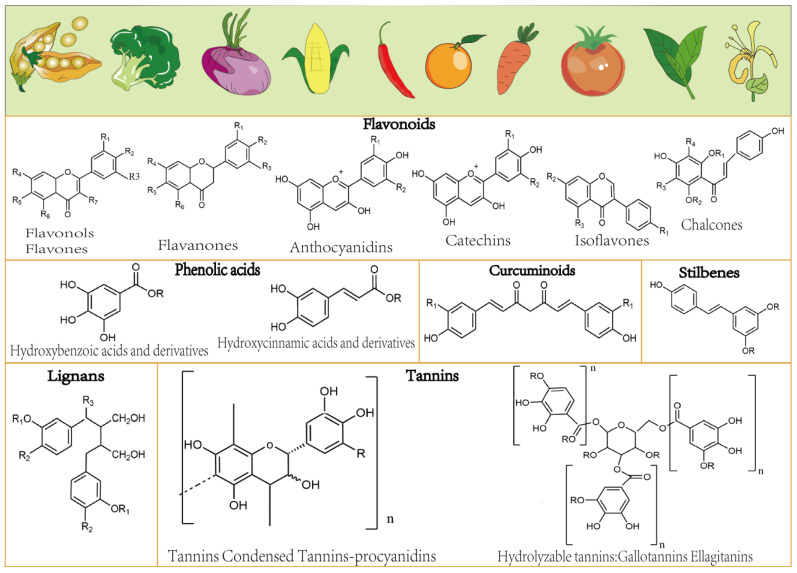

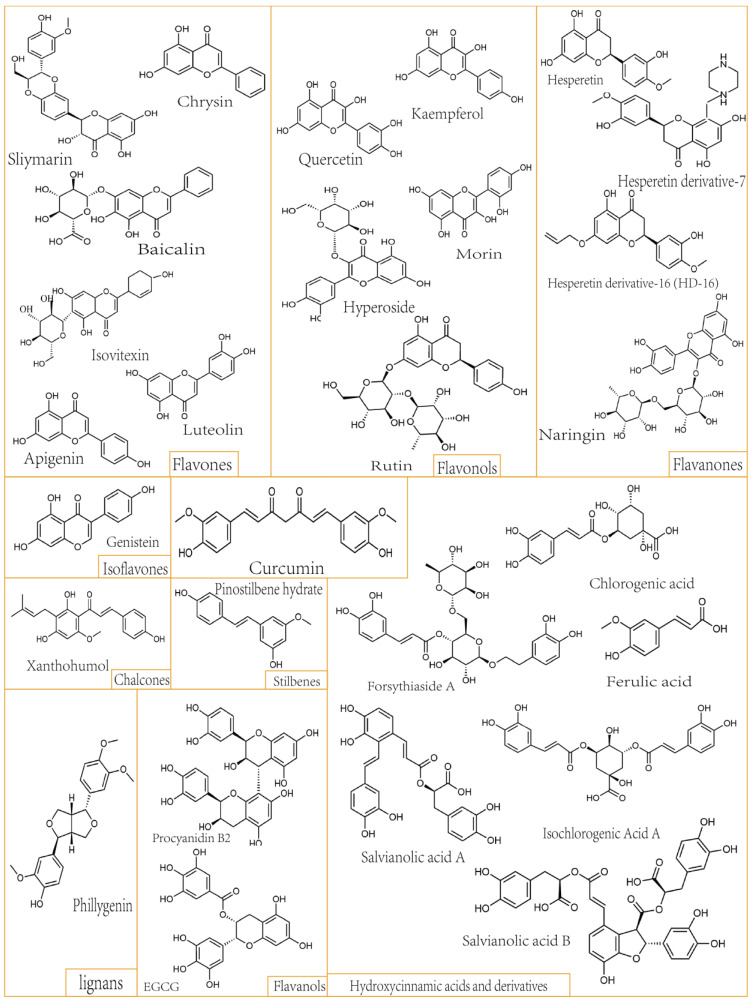
Structure of common phenolic compounds.

Surprisingly, medicinal and food homologs contain a wide range of active substances and nutrients [23]. Some phenols are associated with nutritional and health claims in medicine and food, highlighting this class of ubiquitous, varied, yet unknown compounds. However, health professionals, food technologists, analysts, and consumers do not pay sufficient attention to the phenols in food [24]. These molecules can be divided into phenolic acids, flavonoids, tannins, astragals, and lignans according to the number of phenolic rings and their binding mechanisms [25]. Their classification by molecular mass divides them into polyphenolic monomers, which include both phenolic acid compounds and flavonoids, and tannins formed by the polymerization of monomers [26]. Phenolic acids are divided into two subgroups that have either benzoic acid or cinnamic acid as the parent core, with at least one aromatic ring in its structure and at least one hydrogen substituted by a hydroxyl group on the ring [25]. The subclasses include flavanols, flavanones, flavones, isoflavones, flavonols, and anthocyanidins [27]. Tannins are mainly divided into condensed tannin and hydrolysable tannin, according to their chemical structure. Condensed tannins are oligomers or polymers formed by the polymerization of catechin, epicatechin, gallocatechin, and epigallocatechin in specific ratios; whereas hydrolysed tannins are composed of gallic acid or sugar-containing acids and their derivatives, typified by tannic acid [28]. Lignans are polymers of natural polyphenols that are mainly used in industrial production, and their pharmacological effects have not been extensively studied. Figure 1 shows the classification of dietary polyphenols. Polyphenols are typified by *o*-triphenols and catechols. They are powerful antioxidants and free radical scavengers owing to their easily oxidized *o*-phenolic hydroxyl groups [29] and are associated with a reduced risk of cancer, insulin resistance, cardiovascular diseases, chronic inflammation, and degenerative diseases [30,31,32]. They are receiving increasing attention for the development of potential drugs for the treatment of liver diseases, and many dietary polyphenols have been found to protect against liver fibrosis by inhibiting HSC activity through different signaling pathways. These include apigenin, epigallocatechin-3-gallate (EGCG), quercetin, icaritin, curcumin, and resveratrol [31]. In addition, dietary polyphenols help maintain the homeostasis of human intestinal microorganisms, and the metabolites in microorganisms can regulate the liver and liver fibrosis through signaling pathways. Here, we review previously publications to demonstrate how dietary polyphenols inhibit liver fibrosis through different signaling pathways, gut microbiota, and metabolites. Nanotechnology is also reviewed for its role in improving the poor bioavailability of polyphenol compounds. We discuss current research limitations and propose future directions which would encompass the use of polyphenols to alleviate liver fibrosis and prevent its progression to more serious conditions.

## 2. Dietary Polyphenols Affect Hepatic Fibrosis through Multiple Signaling Pathways

Dietary polyphenols are used in the treatment of liver fibrosis and act through multiple pathways, such as TGF-β/Smad, AMPK/mTOR, Wnt/β-catenin, NF-κB, PI3K/AKT/mTOR, hedgehog pathways and liver fibrosis-related factors. Table 1 lists those dietary polyphenols and their derivatives that have been shown to inhibit HSC activation.

### 2.1. Dietary Polyphenols Reverse Hepatic Fibrosis via TGF-β/Smad Pathway

**Figure 2:**
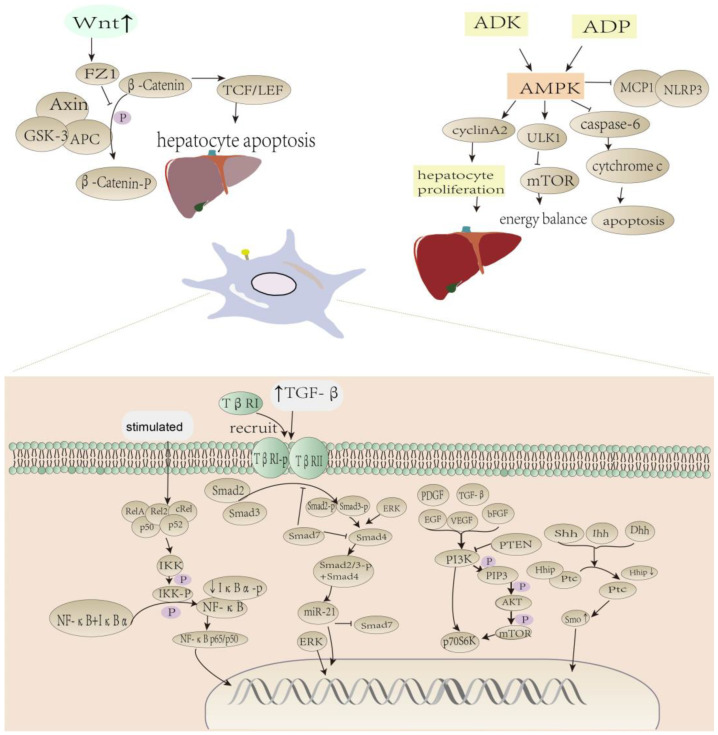
The molecular pathways of liver fibrosis pathogenesis.

TGF-β is the most fibrogenic cytokine in the liver [63,64] and contains three subtypes: TGF-β1, TGF-β2, and TGF-β3 [65]. Under the stimulation of TGF-β, HSCs start to differentiate from a quiescent state into proliferative and fibrotic myofibroblasts that express and secrete α-SMA and collagen types I (CoI-l), CoI-III, and CoI-IV, leading to liver fibrosis [7]. TGF-β can promote the synthesis of ECM proteins, such as proteoglycans, elastin, adhesins, and collagen glycans; reduce the synthesis of degrading proteases; prevent the decomposition of newly synthesized ECM; and destroy the balance between ECM synthesis and degradation [66]. α-SMA invasion is one of the critical hallmarks which can demonstrate HSC activation [67]. Most myofibroblasts express α-SMA and it is postulated that TGF-β1 increases their expression of α-SMA and CoI-l. Blocking or reducing the TGF-β pathway can inhibit or even reverse fibrosis [68], and the direct knockout of TGF-β expression using siRNA has been shown to have anti-fibrotic effects in rat models [69]. In liver fibrosis, TGF-β promotes HSC activation through the TGF-β/Smad signaling pathway [70], and activated receptors are also influenced by Smad-mediated canonical TGF-β signaling mechanisms. Non-Smad signaling pathways, including the mitogen-activated protein kinase (MAPK), extracellular signal-regulated kinases (ERks), c-Jun amino terminal kinase (JNK), p38MAPK, IαB kinase (IKK), phosphatidylinositol-3 kinase (PI3K) and Rho family GTPases, promote the nuclear localization of transcription factors through effects on ERK phosphorylation and Smad2/3 binding to Smad4 [71,72,73]. In the canonical signaling pathway, the activation of HSCs in vitro induces the nuclear translocation of Smad2/3 [71,74]. Activated TGF-β first activates type II receptors on the surfaces of cells, which subsequently recruit TGF-β type I receptor (TβRI), causing the two receptors to form a heterotetramer complex. Type II receptor kinase phosphorylates type I receptors in the GS region, which is upstream of the kinase domain. The receptors then phosphorylate Smad2 and Smad3 to form heterooligomers with Smad4. They are transported from the cytoplasm to the nucleus and regulate miR21 expression in the nucleus. Smad7 acts as an inhibitor, which is also a target of miR21, prevents liver fibrosis by inhibiting Smad2/3. By increasing miR-21 levels, Smad7 is inhibited, which further activates Smad proteins and increases collagen synthesis [71,75,76,77,78,79]. Intracellular Smad7 binds to TβRI and prevents Smad3 phosphorylation and the formation of Smad3-Smad4 heterodimeric complexes, thereby blocking the pro-fibrotic activity in the canonical TGF-β pathway. Additionally, Smad7 binds to DNA and blocks the binding of Smad3-Smad4 heterodimer complexes to DNA in the nucleus [33] (Figure 2).

Dietary polyphenols, such as chlorogenic acid (5-O-cafeoylquinic acid; CGA), are closely associated with the TGF-β signaling pathway [72]. CGA is formed by the condensation of caffeic and quinic acids. It is one of the most abundant phenolic acids in nature and is widely found in fruits, plants, and vegetables such as coffee beans, honeysuckles, tobacco leaves, and kiwi [80]. CGA inhibits the expression of miR-21, α-SMA, and TIMP-1. CGA upregulates Smad7 expression, downregulates p-Smad2, p-Smad3, and p-Smad2/3 levels in vitro and in vivo, and has been shown to inhibit CCl4-induced liver fibrosis in Sprague-Dawley rats [36]. Silymarin is one of the most effective hepatoprotective compounds found in *Silybum marianum* (Milk thistle), belonging to the *Asteraceae* family. Silymarin is a mixture of seven flavonoids, which are called silybin A, silybin B, isosilybin A and isosilybin B, silydianin, silychristin and taxifolin [81,82]. They can reverse hepatic fibrosis in vivo by downregulating TGF-β and inhibiting the secretion of α-SMA and CoI-l [38]. The data aggregated from this study clearly demonstrate that the nanoparticle formulation of silymarin is more effective than the pure silymarin formulation, including controlled release, minimal cytotoxicity, lower dose, targeted entry into hepatic stellate cells for the treatment of hepatic fibrosis [82,83,84]. Curcumin, a polyphenol present in the roots of *curcumin* spp., reduces the phosphorylation levels of JNK and Smad3 and can reverse Smad7 levels [33]. In addition, curcumin remarkably reduced lipid levels, mitigated inflammation and oxidative stress, and improved liver function and hepatic steatosis in patients with non-alcoholic fatty liver disease(NAFLD). Nanocurcumin capsules are a novel drug delivery system that should be studied and applied to boost the clinical efficacy of curcumin [85]. The distribution of free-curcumin solution is widespread throughout the body, and it accumulates in much lower amounts in the liver compared to curcumin-modified nanostructured lipid carriers. Nano-formulation can overcome the problem of curcumin by maximizing its solubility and bioavailability, increasing its membrane permeability, and improving its pharmacokinetics, pharmacodynamics, and biodistribution, thereby improving the effectiveness of curcumin as a drug [86,87,88]. Polyphenols from *Callistephus chinensis* flowers are composed of various flavonoids, which are currently mainly concentrated in flavones (e.g., apigenin, celery glucoside, luteolin, kaempferol-7-O-β-D-glucopyranoside) and flavonols (e.g., kaempferol, quercetin, hyperoside). By inhibiting related proteins in the pathway, downstream target proteins p-ERK1, p-ERK2 and upstream proteins phosphorylation levels of Smad2, p-JNK1, p-JNK2 can be inhibited [64]. In addition, dietary polyphenols such as flavones (chrysin), flavones (luteolin) block TGF-β1-induced activation of HSCs and reverses fibrosis by inhibiting Smad2/3 signaling [40,41]. Luteolin self-nano-emulsifying drug-delivery systems can improve bioflavonoid luteolin dissolution rate and therapeutic effect, as well as protect the liver significantly [89]. Chrysin is a dietary phytochemical that mainly exists in many plant extracts, including propolis, blue passion flower (*Passiflora caerulea*), and honey. Luteolin is abundantly present in many plant extracts, including pepper, chrysanthemum, *Lonicerae japonicae flos*, [90,91]. Therefore, a large collection of dietary polyphenols has been shown to interact with the TGF-β/Smad pathway both as monomers and complexes to treat liver fibrosis.

### 2.2. Dietary Polyphenols Activate AMPK/mTOR-Mediated Autophagic Pathway to Alleviate Liver Fibrosis

Adenosine monophosphate (AMP)-activated protein kinase (AMPK), which senses energy status and controls energy consumption and storage, is an important metabolic regulator [42]. A recent study demonstrated that the AMPK pathway is closely related to liver fibrosis; AMPK can robustly control the proliferation of HSCs, and the lack of AMPK can accelerate the development of liver fibrosis [92]. Studies have shown that adiponectin agonist ADP355, as an agonist of AMPK, alleviates liver fibrosis by promoting AMPK phosphorylation [93]. AMP or adenosine diphosphate (ADP) binding facilitates the phosphorylation of AMPK and increases its antifibrotic activity [94]. The secretion of monocyte chemoattractant protein (MCP)-1 increases the recruitment of inflammatory cells to the site of tissue injury and regulates adhesion molecules and pro-inflammatory cytokines [95]. AMPK can inhibit pro-inflammatory signaling pathways, reduce MCP-1 expression and inhibit NOD-, LRR-, and pyrin domain-containing (3) inflammasome activation to prevent liver inflammation [96]. An autophagic process involves the degradation, recovery, and metabolization of organelles and macromolecules. AMPK is activated in response to metabolic stress, and mTOR is inactivated in response to UNC-51 like kinase protein-induced activation of liver mitochondrial phagocytosis [97]. The activation of AMPKα1 upregulates cyclin A2 transcription and promotes hepatocyte proliferation, finally restoring liver mass after partial hepatectomy. Once the activation of AMPK is reduced, pro-apoptotic caspase-6 cleavage induces the release of cytochrome c, which consequently supports the activation of the executioner caspases and apoptosis in a feed-forward mechanism, leading to liver injury and liver fibrosis [44,98]. Treatment with AMPK activator and caspase-6 inhibitor for two weeks significantly reduced the death of hepatocytes and liver fibrosis. Caspases are related aspartic proteinases that regulate inflammation and cell death [42]. In addition, blocking mTOR and phosphorylating AMPK ameliorates liver fibrosis [46]. A role for AMPK in liver injury treatment may be to maintain energy balance by inhibiting mTOR [99]. AMPK-mTOR is a classic upstream signal-regulation pathway of autophagy. Inhibition of the AMPK signaling pathway leads to a decreased autophagy level. Autophagy is characterized by microtubule-associated protein light chain 3 (LC3) and Beclin1 [100]. Apoptosis and autophagy can be regulated by AMPK, which is located upstream of Beclin1 and B-cell lymphoma-2 (Bcl-2). It has been shown that destroying the Beclin1/Bcl-2 complex increases autophagy in mammals [47]. Although autophagy acts as a double-edged sword in liver fibrosis [99], the role it plays in liver fibrosis remains controversial and needs further study.

The MAPK signaling pathway is closely related to dietary polyphenols [101]. Ferulic acid (FA) is one of the derivatives of cinnamic acid. FA accounts for up to 90% of the total phenolic acids of some fruits and vegetables, including tomatoes, carrots, oranges, and corn [102]. FA has a range of biological activities, including antioxidant, anti-inflammatory, and immune-enhancing properties. FA inhibits hepatic oxidative stress, macrophage activation and HSC activation of hepatocytes via AMPK phosphorylation. The time-dependent effects of FA on P50 and P65 transport from the nucleus to the cytoplasm results in the inhibition of inflammation and the alleviation of fibrosis [44]. The liposomal formulation of ferulic acid overcomes its hydrophobicity significantly and can effectively reach the liver [103]. Hesperetin (flavanones) is mainly distributed in the pericarp of citrus. By activating AMPK/Sirtuins (SIRT3) signaling pathway, hesperetin derivative-16 (HD-16) can increase SIRT3 expression in liver fibrosis [36]. Studies have shown that SIRT3 is generally located in mitochondria and is the downstream signaling target of AMPK [104]. The overexpression of SIRT3 protects liver function and alleviates liver fibrosis. There is evidence that SIRT3 plays a protective role in liver fibrosis by regulating mitophagy [105]. However, SIRT3 knockout impairs the antifibrotic effects of HD-16 by inhibiting the expression of the *α-SMA*, *Col1α1*, *Col3α1*, and *TIMP-1* genes [46]. The treatment of liver fibrosis with curcumin increases p-AMPK levels in a dose-dependent manner and reduces HSC activity [34]. However, it has been shown that combined administration of taurine, EGCG and trihydroxyflavone (genistein) can reduce p-AMPK protein expression and increase p-mTOR protein expression and anti-liver fibrosis. The expression of TGF-β1 in hepatic fibrosis decreased after concurrent use of AMPK inhibitor. The mRNA expressions of *LC3*, *Beclin1* and *mTOR* all decreased; EGCG (flavanols) and genistein (isoflavones) are present in tea and soybean as characteristic components [27,106]. However, research on the role of polyphenols in alleviating fibrosis through the AMPK signaling pathway is poor and controversial at present, and further studies are needed.

### 2.3. Dietary Polyphenols Reverse Wnt/β-Catenin Pathway in Hepatic Fibrosis

An inhibition of the Wnt/catenin signal (canonical) may limit the activation of HSCs by maintaining their static state [49]. It is reported that Wnt/β-catenin activation of the signal can promote liver fibrosis [107]. During liver fibrosis, the Wnt signaling pathway is activated, and some Wnt signaling pathway elements are upregulated. Silencing β-catenin inhibits CoI-l/III synthesis in HSCs, which is the core component of the Wnt/β-catenin pathway. Activated HSCs induce Wnt ligands (Wnt3a, Wnt4, Wnt5a, and Wnt10b) and Wnt receptors (Frizzled1 (Fz1), Frizzled2 (Fz2). In addition, canonical (*β-catenin*) and non-canonical (*Wnt4* and *Wnt5a*) *Wnt* genes were increased in activated HSCs [11]. A destructive complex of Axin, adenopolyposis coli (APC), and glycogen synthase kinase 3 (GSK-3) forms under unstimulated conditions. Cytoplasmic β-catenin levels are kept lower by phosphorylation of GSK-3. It is ubiquitinated and targets the proteasome for degradation when it becomes phosphorylated. Axin, APC, and GSK-3 complexes are inhibited when Wnt binds to the receptor complex, blocking the phosphorylation of GSK-3 to β-catenin. Low-levels of phosphorylated β-catenin accumulate in the cytoplasm and are transferred to the nucleus, where they regulate the expression of target genes by cooperating with the T-cell-specific transcription factor/lymphoid enhancer binding factor 1 (TCF/LEF) family of transcription factors [49,108]. TCF/LEF is a type of transcription factor with a dual regulatory role in the nucleus, and when combined with β-catenin, it promotes the transcription of downstream target genes and hepatocyte apoptosis [109]. The activation of target genes is triggered by the Wnt proteins binding to the Frizzled family of receptors, illustrated in Figure 2 [35].

Dietary polyphenols such as pinostilbene hydrate (3,4’-Dihydroxy-5-methoxy-trans-stilbene hydrate, PSH) are available; it is a non-flavonoid natural methylated derivative of resveratrol. There is some evidence that PSH can significantly reduce nuclear β-catenin, β-catenin nuclear translocation, and TCF activity. PSH decreased Col1α1 and the expression of α-SMA was blocked when WIF1 (as a Wnt signal inhibitor) was silenced. Complexes consisting of APC, AXIN1 and GSK3β are known to downregulate β-catenin stability and induce its degradation. PSH resulted in the inactivation of the Wnt/β-catenin signal, decreased TCF activity and β-catenin nuclear migration, and increased WIF1, GSK3β, APC and P-β-catenin levels [48]. Hesperetin deactivative-7 decreased β-catenin and downstream proteins, such as cyclin1 and C-myc, which reduced liver fibrosis [49]. A flavonoid compound called baicalin is mainly found in the dry roots of Scutellaria baicalensis [110]. Baicalin reduces BDL-induced HSC activity by inhibiting Peroxisome proliferator-activated receptor-γ(PPAR-γ) through the Wnt pathway, thereby alleviating liver fibrosis [111]. Compared with baicalin alone, nanoliposomes loaded with baicalin had a greater effect on mice induced with NAFLD from a choline-deficient diet [112]. The typical Wnt/β-catenin signaling pathway is a complex, controllable molecular mechanism that regulates key physiological and pathological processes such as cell proliferation, differentiation, and polarity in multicellular organisms. Although the Wnt/β-catenin pathway is activated during HSC activation, its role in fiber formation remains controversial [113]. Hence, it is necessary to conduct further studies in order to clarify the relationship between dietary phenolics, the Wnt/β-catenin pathway and liver fibrosis.

### 2.4. Polyphenols Inhibit the NF-κB Pathway in Liver Fibrosis

It is worth noting that nuclear factor kappa-B (NF-κB) activity is crucial to the expression of anti-apoptotic proteins [114]. The activation of NF-κB accelerates liver injury and inflammation, followed by massive hepatocyte death and the inflammatory activation of HSCs leading to liver fibrosis [51]. The NF-κB family of transcription activators includes RelA, RelB, and cRel, and two family members (p50 and p52) forming heterodimers with transcriptional active proteins. Additionally, these simulations predicted that increased RelA activity might decrease cRel activity by competing for p50 [115,116]. These transcription factors may regulate inflammation and apoptosis primarily through the NF-κB/IκBα signaling pathway [117]. The transcription factor of NF-κB binds to the nuclear factor κB (IκB) protein inhibitor during the resting state condition, keeping the transcription factor localized in the cytoplasm [118,119,120]. When hepatocytes are stimulated, this prompts IKK activation and phosphorylation, which later promotes IκBα phosphorylation, resulting in the dissociation of the IκBα-NF-κB complex [119]. IKK activation, IκBα phosphorylation and subsequent rapid degradation by the targeting proteasome, resulting in NF-κB p65 subunit activation and translocation from the cytoplasm to the nucleus, and thus IκBα is used to reflect NF-κB translocation [121,122]. NF-κB translocates to the nucleus upon activation and stimulates pro-inflammatory genes such as *interleukin-6* (*IL-6*), *Tumor necrosis factor* (*TNF-α*) and *Inductible Nitric Oxide Synthase* (*iNOS*) [50]. This activation occurs mainly through phosphorylation and degradation of the repressor IκBα, releasing the cytoplasmic dimer NF-κB p65/p50. The cytoplasmic dimer binds to DNA and stimulates the transcription of the target gene in the nucleus [123]. In addition, there is a crucial role for GSK-3 in the regulation of proinflammatory and anti-inflammatory factors, mainly affecting the NF-κB receptor [99]. GSK3 signaling is critical for the production of proinflammatory cytokines such as interleukin (IL-1β), IL-6, interleukin-12 (IL-12) and TNF-α; the anti-inflammatory cytokine interleukin-10 (IL-10) in innate immune cells is differentially regulated [99]. It is intriguing to speculate that NF-κB-mediated anti-apoptotic responses to TNF-α depend on GSK-3β function [45,124], as illustrated in Figure 2.

Consumption of dietary polyphenols suppresses the pro-inflammatory process that develops in liver diseases by downregulating the NF-κB pathway [125]. FA has numerous beneficial biological and pharmacological effects. FA targeting accelerates GSK-3β to repress the binding ratio of p-NF-κB to CSK-binding protein (CBP) and CREB (Ser133) to CBP, thereby increasing the anti-inflammatory factor IL-10 and decreasing the pro-inflammatory factors IL-1β, IL-6, IL-12 and TNF-α [45]. The salvianolic acid A (sal-A) plant is mostly found in *Salvia miltiorrhiza*, it has been shown that sal-A can decrease or increase the levels of NF-κB in the nucleus and cytoplasm, respectively; it inhibited both NF-κB and IκBα dimer disaggregation in the cytoplasm, thereby alleviating NF-κB translocation into the nucleus [52]. An isochlorogenic acid is a polyphenol made up of two molecules of caffeic acid and one molecule of quinic acid [126]. Isochlorogenic acid A significantly decreased NF-κB p65 expression in the nucleus and increased NF-κB p65 expression in the cytoplasm, reducing the phosphorylation of IκBα and activation of NF-κB [54]. Xanthohumol is a hop-derived chalcone that has been widely examined for its health-protecting properties [127]. Xanthohumol inhibits MCP-1 and interleukin-8 (IL-8) by decreasing NF-κB activity. Fibrosis in nonalcoholic steatohepatitis is associated with an increase in MCP-1 and IL-8 [55]. Therefore, studies have shown that dietary polyphenols play an important role in inhibiting the activation of transcription factor NF-κB and HSC.

### 2.5. PI3K/AKT/mTOR Pathway Effects of Dietary Polyphenols

The PI3K/protein kinase B (AKT)/mammalian target of rapamycin (mTOR) pathway plays an indelible role in cell activation, proliferation, differentiation, and survival [10]. Several growth factors, such as PDGF, TGF-β, epidermal growth factor (EGF), vascular endothelial growth factor (VEGF), and basic fibroblast growth factor (bFGF), are implicated in the cellular process which activates PI3K and AKT [10,128]. PDGF is the most powerful mitogen of HSC, which can activate the PI3K/AKT/70-kDa ribosome S6 kinase (p70S6K) signaling pathway, and regulate HSC proliferation and migration [41]. PI3K/AKT/mTOR signaling facilitates the proliferation, activation and synthesis of ECM by HSCs [129]. In the fibrosis model experiment, the phosphorylation of AKT was significantly increased [41,56]. As a PI3K inhibitor, HS-173 relieves activated HSCs associated with liver fibrosis by blocking the PI3K/AKT pathway [129,130,131]. PI3K is a heterodimeric protein consisting of a regulatory subunit of 85 kDa and a catalytic subunit of 110 kDa that binds to the PDGF receptor and is activated through phosphorylation [132,133]. PI3K is activated and converted to phosphatidylinositol 3,4-triphosphate (PIP3), which is actually phosphorylated by PI3K [95,107]. As a result of PI3K activation, phosphorylated inositol lipids are generated, which function as essential second messengers for intracellular signaling. As a downstream target in the PI3K pathway, phosphorylated inositol lipid binds to Akt and induces its translocation to the plasma membrane [132,133]. The phosphorylation of Akt by PIP3 facilitates a variety of signal-transduction processes related to apoptosis. PIP3 promotes the aggregation and activation of Akt, which directly phosphorylates mTOR. Through active mTOR phosphorylation, downstream protein P70S6K promotes mRNA synthesis, translation, transcription, and growth and proliferation of cells [119,134]. PI3K also activates p70S6 kinase (p70S6K), a ribosomal 70-kDa protein affected by mitogens, growth factors, and hormones, downstream of Akt [132,133]. It has been demonstrated that CCl4 activates PI3K/AKT signaling by significantly increasing the phosphorylation of mTOR, PI3K, and AKT in the hepatic fibrosis cells, as illustrated in Figure 2 [119].

Luteolin (3,4,5,7-tetrahydroxyflavone) is a flavone mainly distributed in broccoli, celery, chrysanthemum flowers, onion leaves, broccoli, carrots, peppers, parsley, and thyme [135]. Luteolin reduces PDGF-induced AKT (Ser473) phosphorylation and downstream mTOR molecules and mTOR substrate p70S6K in a dose-dependent manner. Luteolin also reduces TGF-β1-induced AKT signaling [41]. Naringin (4′,5,7-trihydroxyflavonone-7-rhamnoglucoside) is a natural flavone glycoside extracted from grapefruit and oranges. Naringin regulates cell survival by blocking the PI3K/AKT signal to relieve liver fibrosis [56]. Sal-A reduces the stimulation of the PI3K/AKT/mTOR signaling pathway, inhibits the stimulation of HSCs and reduces the deposition of ECM [53]. Rutin (*Flos Sophorae Immaturus*) and curcumin induce NHSC (non-chemically induced HSC) autophagy by stimulating fatty acids by regulating the PI3K/AKT/mTOR pathway, resulting in the inhibition of NHSC activation [57]. It is reported that isovitexin (IVT) is derived from H. sibthorpioides, a plant of the Umbelliferae family that may treat diseases of the liver, such as cirrhosis, liver fibrosis, and jaundice [136]. PTEN (phosphatase and tensin homolog deleted on chromosome ten) negatively regulates PI3K expression, which is a downward regulation during the progression of fibrosis and a positive regulation during recovery. Decreased PTEN expression may stimulate stellate cell activation via the activation of the PI3K-Akt-mTOR pathways, and these regulatory effects are closely associated with autophagy. IVT dramatically augmented the apoptosis rate of HSCs, decreased the cell viability, and inhibited Col-I, Col-III and α-SMA mRNA levels and protein expressions, suggesting the inhibition of HSC activation and increased autophagy. A significant increase in p-PTEN levels and a reduction in levels of p-PI3K, p-Akt, and p-mTOR in the liver tissues of mice were observed following IVT treatment. When a PTEN inhibitor or PI3K activator was treated, the effect of IVT on autophagy and HSCs was impaired, suggesting that the PI3K-Akt signaling pathway is the hub of IVT regulation of autophagy and HSC activation [136]. A small number of polyphenol compounds are also effective in the treatment of liver fibrotic diseases by activating the pathway of PI3K/AKT/mTOR. PI3K/AKT/mTOR regulates autophagy and reverses hepatic fibrosis.

### 2.6. The Effect of Dietary Polyphenols on the Hedgehog Signaling Pathway

The hedgehog signaling (Hh) pathway is a highly conserved signaling pathway in cells and regulates various diseases [137]. Hh signaling is thought to be inactivated in healthy adult livers because Hh ligands are rarely expressed in mature hepatocytes [138,139]. Hh signaling contributes in activated HSCs and liver fibrosis [140]. Studies have shown that the upregulation of Hh signaling promotes the development of hepatic fibrosis and inhibits Hh signaling to inhibit hepatic fibrosis [141]. Hh signaling promotes the transition of quiescent HSCs to fibroblasts. Researchers have found that quiescent HSCs produce large amounts of Hhip (an inhibitor of hedgehog) and prevent its binding to Patched (Ptc) receptors [43,138]. Hhip is rapidly downregulated when HSC is activated and hedgehog target genes (such as *GLI family zinc finger 2* (*Gli2*)) are increased [140]. The canonical pathway involves Hh ligands (Shh, Ihh and Dhh) binding to the transmembrane receptor Ptc, causing Ptc to release Smoothened (Smo, a G protein coupled receptor) [141,142,143]. The released Smo accumulates in the primary cilium, which conduces to the nuclear localization of glioma-associated oncogene homology (Gli) transcription factors, and there are three Gli transcription factors (Gli1, Gli2, and Gli3). Gli2 is described as the main activator of Hh signaling, whereas Gli3 is responsible for the inhibitory function of Hh signaling [43,141,142,143]. Gant61 (a Gli1/2 transcription factor inhibitor), Gant61 alleviates liver fibrosis by decreasing the number of HSCs and decreasing the mRNA and protein levels of Smo, Gli1, Gli2 [141]. Gli2 or Gli3 binds to DNA and then regulates the transcription of many Hh target genes in the nucleus. In the noncanonical signaling pathway, Ptc was shown to regulate the cell cycle through cyclin B1, without the need for Smo and Gli transcription factors. Downstream effects of Smo have also been shown to be mediated by the sensitization of small GTPases independent of Gli transcription factor as illustrated in Figure 2 [141,142,143,144].

There are about 100 medicinal plants containing quercetin, which is found in a number of foods. Quercetin (3,3,4,5,7-pentahydroxyflavone) is a flavonoid and has good safety and bioavailability when used as a supplement. It can relieve Shh, Ihh, and hedgehog ligand-receptor Ptc1 expression induced by thioacetamide (TAA), reduce the level of Smo and Ptc1 mRNA, and relieve liver fibrosis [145]. Quercetin could be selectively delivered to activated HSCs using multifunctional integrin-targeted nanoparticles [146]. Liver fibrosis is associated with *HIF-1*, a gene that is targeted by Hh signaling. HIF-1α is highly expressed in fibrotic tissues. The induction of liver fibrosis is alleviated by flavanols that are derived from proanthocyanidin dimers, such as procyanidin B2 [59]. In the absence of Hh ligands, Ptc1 reduces the expression of Smo by inhibiting this pathway. Hesperetin derivatives (HDs) regulate and enhance the expression of Ptc1 in HSCs to enhance liver protection [60]. Salvianolic acid B (SalB) caused a significant reduction in Shh, Ptc1, Smo and Gli1 mRNA levels in liver tissue, thereby inhibiting HSC activity [61]. Moreover, studies have shown that giving SalB can induce demethylation of DNA methyltransferase 1 (DNMT1) to regulate the Ptc1 gene. Ptc1 hypermethylation is associated with the activation of fibroblasts in liver and the persistence of liver fibrosis. SalB can silence DNMT1 and demethylate Ptc1, thereby inhibiting the Hh signaling pathway to relieve the activated HSC [147]. It appears that SalB mesoporous silica nanoparticles, rhodamine B, is more effective than free SalB at increasing cellular drug uptake, drug bioaccessibility, and antiROS and hepatic fibrosis efficacy [148].

### 2.7. Role of Dietary Polyphenols on Liver Fibrosis-Related Factors

As a key transcription factor mediated by ROS, nuclear factor-erythroid 2-related factor 2 (Nrf2) plays an important role in protecting cells from oxidative stress by promoting the expression of many antioxidant genes, such as *heme oxygenase-1* (*HO-1*) and *NAD* (*P*) *H*: *quinone oxidoreductase* (*NQO1*), *glutathione cysteine ligase modified subunit* (*GCLM*) and *glutathione cysteine ligase catalytic subunit* (*GCLC*) stimulate ring protection genes. Nrf2 induction is a potential target for the alleviation of toxic liver injury and fibrosis [149]. In vitro, *Nrf2* gene transfer to human and rabbit aortic smooth muscle cells can inhibit the secretion of MCP1, Nrf2-dependent HO-1 expression can inhibit TNF-α stimulated NF-κB and the secretion of MCP-1 in human umbilical vein endothelial cells [150]. Studies have shown that the activation of Nrf2 alleviates liver fibrosis and nonalcoholic steatohepatitis [151]. Normally, Nrf2 is in a bound state with keapl (as the main inhibitor of Nrf2) in the cytoplasm [152]. When oxidative stress occurs, it will dissociate in the form of dimers and combine with antioxidant components, participating in the synthesis of antioxidant enzymes and phase II detoxification enzymes, and defend against the progress of liver fibrosis by increasing the antioxidant capacity of the liver. HO-1 and NQO-1 are antioxidant defense genes completely dependent on Nrf2 [52,152]. Silibinin/bovine serum albumin (SIL/BSA) nanoparticles exhibited antioxidant effects against intracellular oxidative stress via the upregulation of the Nrf2/antioxidant responsive element (ARE) pathway, decreasing ROS and regulating antioxidant enzyme reactivity. There was a significant reduction in acetaminophen and lipopolysaccharides(LPS)/D-GalN-induced acute liver injury in mice when SIL/BSA nanoparticles were compared to SIL formulation since SIL/BSA nanoparticles presented better biocompatibility and more liver distribution [39]. Morin (3,5,7,2′,4′-pentaxyl flavone), a natural flavonol, is extracted from mulberry leaves and has inhibitory effects on LX-2 cells (a hepatic stellate cell) in vitro by reducing the expression of GFAP (activated HSC marker) [35,153]. It has been reported that morin acts as an exogenous agonist of Nrf2 and promotes its nuclear translocation to exert its biological effects [154]. In the morin-treated group, Nrf2 and its downstream products, including NQO1 and HO-1, were significantly elevated, indicating that morin plays a role in alleviating liver fibrosis through the Nrf2 pathway [153]. The expression levels of HO-1, NQO-1 and GCLC were increased dose-dependently by sal-A. Improvement in the synthesis and activation of Nrf2 in liver tissue to prevent oxidative damage thus alleviated CCl4-induced liver damage [52].

## 3. Dietary Polyphenols Affect Gut Microbiota Composition in Liver Fibrosis 

### 3.1. Effect of Dietary Polyphenols on Intestinal Microbial Composition

The liver and gut microbes are anatomically and physiologically connected by the portal vein and interact with each other [155,156,157]. Studies have shown that intestinal microbes in the gastrointestinal tract can remotely regulate a variety of organ injuries, especially in the liver [158]. In addition, the occurrence of liver fibrosis is followed by the imbalance of intestinal micro-homeostasis, the decrease in intestinal microbial diversity and richness, the increase in potential pathogenic bacteria and the reduction in beneficial bacteria [159]. At the phylum level, the change in the *Firmicutes/Bacteroidetes* ratio is an important indicator of a change in the intestinal flora structure, which can indicate liver fibrosis [157,160]. Research shows that an increase in *Ruminococcus* abundance is independently related to fibrosis [161]. It seems that polyphenols can change the intestinal microecology, affect the total number of beneficial bacteria in the intestine, and bring positive intestinal health benefits [162]. It is reported that dietary polyphenols can treat liver immune diseases [163], nonalcoholic fatty hepatitis, nonalcoholic fatty liver disease and other related liver diseases by regulating intestinal microorganisms [164]. Because inflammation is accompanied by various stages of liver fibrosis, polyphenols can stimulate *Firmicutes phylum*, *Bifidobacterium* spp., *Akkermansia* spp., *Roseburia* spp. and *Faecalibacterium* spp. It is beneficial to the growth of bacteria, can provide anti-pathogenic and anti-inflammatory effects, and inhibit the growth of pathogenic bacteria *Clostridium* spp. (*Firmicutes phylum*) [165]. Phillygenin (PHI) is one of the main lignans in weeping forsythia capsules [149]. PHI increased the abundance of *Ruminococcaceae_UCG-014* and *Lactobacillus*, and decreased that of [*Eubacterium*]*_coprostanoligenes_group*, in which *Lactobacillus* has a liver protection effect. It can be concluded that PHI can alleviate CCl4-induced liver injury and liver fibrosis by regulating intestinal microorganisms [62,166]. In addition to regulating intestinal flora and inhibiting inflammation to alleviate liver fibrosis, dietary polyphenols can also be directly metabolized by colonic microorganisms. Its metabolites may not only balance the homeostasis of intestinal flora, but also have stronger bioactivity of metabolites than native phenolic compounds, and enhance the absorption and bioavailability of the body [167,168,169]. Therefore, dietary polyphenols may be a latent intervention for improving intestinal microbial disturbance in patients with liver fibrosis.

### 3.2. Polyphenols Regulate Liver Fibrosis by Influencing Microbial Metabolites

**Figure 3:**
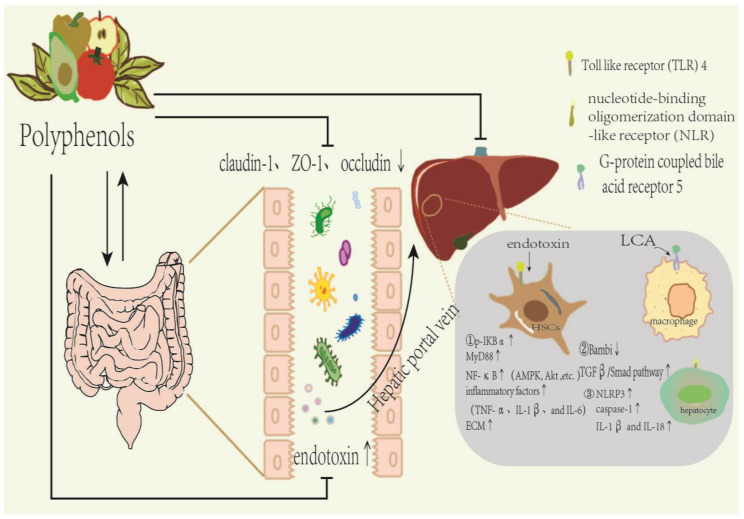
Dietary polyphenols act on liver fibrosis by regulating intestinal microbiota and metabolites.

There is strong evidence that gut microbial metabolites are involved in inflammation and liver fibrosis pathways [156]. Gut microbial metabolites such as bile acids, LPS, endogenous ethanol and short-chain fatty acids (SCFA), etc., act as messengers across the intestinal barrier into the liver to activate inflammation-related signals and regulate fibrosis progression [159,168]. Liver immune cells are activated and induced by the innate immune response of the liver to produce inflammatory cytokines that drive the production and progression of liver fibrosis [156,158,170,171,172]. Endotoxin interacts with hepatic CD14, Toll-like receptors(TLR4) and other receptors to enhance the phosphorylation and degradation of IκBα in the cytoplasm, leading to the activation of NF-κB [168]. It also enhances myeloiddifferentiation factor88 (MyD88) recruitment through the activation of TLR4 and accelerates nuclear transcription of NF-κB to activate the TLR4-MyD88-NF-ΚB signaling pathway. The upregulation of levels of inflammatory cytokines (e.g., TNF-α, IL-1β and IL-6) stimulates extracellular matrix synthesis in HSCs and causes or exacerbates liver fibrosis [159,168,173]. LPS regulates innate immunity by activating TGF-β signaling progression via TLR4 in the HSC [174]. TLR4 stimulation promotes liver fibrosis by downregulating Bambi, an endogenous decoy receptor for TGF-β, and upregulating the TGF-β/Smad signaling pathway. In addition to enhancing exposure to TGF-β derived from Kupfer cells, LPS also heightens receptor sensitivity to induce inflammation in the liver and facilitate the development of liver fibrosis [159,168,170]. Furthermore, primary bile acids are converted to secondary bile acids by gut microbes, and the flora act as important players in bile acid synthesis, modification, and signaling. Lithochalic acid (LCA) is a secondary bile acid that inhibits inflammation and ECM synthesis by activating bile acid receptor 5 (TGR5), which binds G-proteins on the surface of macrophages and HCS. LCA reduces IL-1β, TNF-α, caspase-1 and IL-22 levels, inhibits the TLR4/NF-κB pathway and NLPR3 inflammation to improve liver inflammation. In addition, LCA reduces the influence of HSC on TGF-β signal sensitivity and promotes ECM degradation [175]. Another metabolite of intestinal bacteria, SCFA, not only has the effect of inhibiting inflammation, but may also accelerate the differentiation of naive T cells into Th1 and Th17 cells, so as to improve the body’s immunity against pathogens [176], as illustrated in Figure 3.

Many studies have reported that dietary polyphenols can decrease liver inflammation and liver fibrosis by regulating the regulation of the liver-related inflammatory signaling pathway using the metabolites of intestinal microorganisms. For example, baicalin has potential therapeutic effects on liver and intestinal diseases by regulating farnesoid X receptor (FXR) and TGR5 to mediate bile acid crosstalk related to intestinal microorganisms. Baicalin helps to increase the number of bacteria producing SCFA, reduces the phosphorylation of PI3K, AKT and mTOR, and lowers the level of IL-17 to inhibit liver fibrosis [111,177]. Chlorogenic acid treatment of liver fibrosis is mainly through improving the intestinal microbial composition, increasing the level of SCFA, and inhibiting inflammatory factors [178]. It mainly inhibits the activation of the TLR4 signaling pathway, including the reduction of TLR4, MyD88 expression, the increase in Bambi and IKB proteins, the nuclear translocation of NF-κB and p-IKB-reduction [37]. In addition to chlorogenic acid, isochronogenic acid A (ICQA) reduces liver fibrosis and the expression of high-mobility group box 1 (HMGB1) and TLR4, alleviates NF-κB p65 nuclear translocation, and inhibits p-IKB expression. It is suggested that ICQA protects against liver fibrosis induced by CCl4 by inhibiting the HMGB1/TLR4/NF-κB signaling pathway [54]. PHI reduces the level of LPS in serum and of inflammatory factors (IL-1β, IL-6, and TNF-α) in liver tissue [22]. It promotes the production of SCFA, regulates the imbalance of bile acid, and may alleviate liver fibrosis [166]. Forsythiaside A (FTA), as a polyhydroxy structural compound, reduces LPS, macrophage inflammatory protein-1 and TNF-α in serum. Additionally, FTA increases the abundance of SCFA-producing bacteria and SCFA products, thus protecting the liver [22]. It can be concluded that the interaction between dietary polyphenols and the intestinal microenvironment is essential for the clinical treatment of liver fibrosis.

## 4. Conclusions

In recent years, in spite of an increasing morbidity and mortality rate in relation to liver fibrosis, there are no effective drugs available to treat it, and clinically used drugs for treating liver fibrosis have poor efficacy or side effects. Fibrosis-specific drugs are urgently needed. It can develop into more severe liver cirrhosis or even cancer if left untreated. Dietary polyphenols have anti-inflammatory and antioxidant properties, and they have been reported to inhibit TGF-β/Smad, Wnt/β-catenin, NF-κB, PI3K/AKT/mTOR, hedgehog pathway, or activate the AMPK/mTOR pathway to reverse liver fibrosis. In addition, they can regulate gut microbiome composition to alleviate liver fibrosis. These metabolites of gut microbes regulate various signaling pathways and the immune system indirectly to reduce liver fibrosis through the gut–liver axis. Currently, there are still many disadvantages associated with dietary polyphenols in the treatment of liver fibrosis, which need to be addressed. First, their poor bioavailability greatly limits their anti-fibrotic and anti-inflammatory effects, despite their strong biological activity and ability to be partially metabolized by gut bacteria. However, the emergence of nanomedicine, which is stable and scalable has shown high translational potential. It may reduce the toxic side effects of polyphenol compounds. The advantages of nanotechnology for polyphenol compounds include controlled release, lower dose, remarkable hepatic-targeting effect, superior pharmacokinetic properties, and superior biosafety, thus reducing the damage to other tissues. In general, polyphenolic compounds used in the treatment of hepatic fibrosis, such as milk thistle, curcumin, baicalin, etc., are of definite therapeutic benefit. More large-scale, long-term, high-quality clinical trials are necessary in the future to assess the efficacy and safety of polyphenols in the treatment of liver fibrosis in order to promote drug development and clinical application. Secondly, there are occasionally toxic side effects associated with polyphenols, and further research is needed to understand their mechanism of action against liver fibrosis. Furthermore, there are significant differences between cell and animal experiments and human clinical application. The current level of research is limited to cells or animals. Polyphenols in the diet are difficult to achieve in clinical trials and need to be improved. Finally, there are a sea of polyphenol compounds in the diet and a small part of the polyphenol compounds have been shown to treat liver fibrosis. In addition, it is difficult to explain which polyphenol compounds are beneficial to liver disease in dietary polyphenols. To treat liver fibrosis, dietary polyphenol compounds must be developed in the future. Due to the multiple pathways involved in hepatic fibrosis, a treatment targeting multiple factors is expected to address the driving factors. In the future, in order to better understand the relationship between dietary polyphenols and gut microbiomes and signaling pathways, we look forward to designing more systematic experimental models to investigate the anti-fibrosis and liver-protective effects of dietary polyphenols. In clinical studies, dietary polyphenols that are known to have therapeutic effects need to be evaluated for their dose, toxicity, and bioavailability to contribute to the treatment and improvement of liver fibrosis.

## Figures and Tables

**Table 1:** Dietary polyphenols function as active substances in anti-fibrosis treatments. ↑ increase, ↓ decrease.

Polyphenols/Polyphenol-Rich Plants	Categories/ Identified Polyphenols	Examples of Dietary Sources	Experimental Models	Dosage	Duration	Effects	References
Forsythiaside A	Phenolic acid	Forsythiae Fructus	CCl4-induced mouse	15, 30, 60 mg/kg	4 weeks	claudin-1↑, ZO-1↑, inclusion↑, LPS↓, MIP-1↓	[22]
Callistephus Chinensis flower	Polyphenol		Rat model (CCl4-induced)	50, 100 mg/L	6 weeks	TGF-β1↓, Smad2↓, P-ERK1↓, P-NK1↓	[26]
Curcumin	Polyphenol	Roots of *curcumin* spp.	Rat model (CCl4-induced)	100 mg/kg	4 weeks	JNK↓, Smad3↓, Smad7↑	[33]
			Primary rat HSCs	20 µM	24 h	α-SMA↓, Col1α1↓ PPAR↑, AMPK↑	[34]
			HSCs	25 µM	24 h	TLRs↓, MyD88↓, NF-κB↓, TNF-α↓, IL-1β↓	[35]
Chlorogenic acid	Phenolic acids	Coffee beans, honeysuckle, tobacco leaves and kiwi	hepatic stellate LX2 cell line	20, 40, 80 µg/mL	24 h	miR-21↓, α-SMA↓, TIMP-1↓, Smad7↑, MMP-9↑	[36]
			Rat model (CCl4-induced)	15, 30, 60 mg/kg	4 weeks	miR-21↓, α-SMA↓, TIMP-1↓, TGF-β1↓ Smad7↑, MMP-9↑	
			Rat model (CCl4-induced)	60 mg/kg	8 weeks	TLR4↓, MyD88↓, NF-κB↓, p-IκBα↓ Bambi↑, IκB α↑	[37]
Silymarin	Flavonoid	Silybum marianum	CCl4- or BDL-induced fibrosis			TGF-β↓, α-SMA↓, collagen I↓	[38]
			LPS/D-GalN induced liver injury			(Nrf2)/antioxidant responsive element (ARE) pathway↑ caspase 9/3 related apoptosis pathway↓	[39]
Chrysin	Flavonoids	Propolis, blue passion flower (Passiflora caerulea), and honey	Rat model (CCl4-induced)	50, 100 and 200 mg/kg	2 weeks	α-SMA↓, TGF-β1↓, Smad 2/3↓	[40]
Luteolin	Flavonoids	Pepper, chrysanthemum, Lonicerae japonicae flos	rat models CCl4, dimethylnitrosamine (DMN) and bile duct ligation (BDL)			p-AKT↓, p-Smad2↓	[41]
			Primary HSCs and HSC-T6 cells	TGF-β1 (2 ng/mL)	2 h	a-SMA↓, collagen I/III↓, AKT↓, Smad2/3↓, TGF-β1↓	
Baicalin	Flavonoids	Roots of Scutellaria baicalensis	BDL-induced	67.5–270 μM		PPAR-γ↓, Wnt↓	[42,43]
				25–100 mg/kg		SCFA↑, regulating FXR and TGR5 receptor↑, PI3K↓, AKT↓, mTOR↓, IL-17↓	
Ferulic acid	Phenolic acid	Tomatoes, carrots, oranges, and corn	RAW 264.7 cells and LX-2 cells	50, 100, 200 μM And 12.5, 25, 50 μM	24 h	Acta2↓, Col1a1↓, p-Smad↓, p-Smad3↓, p-AMPK↑	[44]
			(mice) CCl4-induced	25, 50 and 100 mg/kg		ALT↓, AST↓, TGF-1β↓, Acta2↓, NOX2↓, SOD↑, AMPK↑, ERK1/2↑	
			lipopolysaccharide (LPS)-induced cellular ALI models	6, 12 mg/kg	6 day	GSK-3β↑, CREB (Ser133)↑, IL-10↑, p-NF-κB↓, IL-1β↓, IL-6↓, IL-12↓, TNF-α↓	[45]
Hesperetin derivative-16 (HD-16)	Flavonoids	Pericarp of citrus	LX-2 cells (human immortalized HSCs)	4, 8, and 16 μM		α-SMA↓, Col1α1↓, Col3α1↓, TIMP-1↓, TNF-α↓, IL-1β↓, IL-10↑, IL-13↑, SIRT3↑	[46]
			CCl4-induced mouse	25 mg/kg, 50 mg/kg, 100 mg/kg		ALT↓, AST↓, ALP↓, α-SMA↓, Col1α1↓, TNF-α↓, IL-1β↓, IL-10↑, IL-13↑, SIRT3↑	
Salvianolic acid A	Phenolic acid	Salvia miltiorrhiza	CCl4-induced rats	5, 15 mg/kg	6 weeks	p-AKT↓, p-mTOR↓, p-p70S6K1↓, caspase 3↓, Bax↓, α-SMA↓, PDGF-β↓, Desmin↓, Vimentin↓, TGF-β1↓, Bcl-2↑	[47]
Pinostilbene hydrate	Phenolic acid		Primary HSCs	20 μM, 80 μM	48 h	Wnt/β-catenin↓, WIF1↑, GSK3β↑, APCP↑, β-catenin↑	[48]
Hesperetin derivative-7	Flavonoids		CCl4-induced mouse	50, 100, 200 mg/kg	4 weeks	SMA↓, collagen I↓, β-catenin↓, c-myc↓, cyclind1↓,	[49]
			HSC-T6 cell line	12.5, 25, 50, 100, 200 μM	48 h	p-smad3↓, smad4↓ α-SMA↓, β-catenin↓, cyclind1↓, c-myc↓	
Morin	Flavonoids	Mulberry leaves	diethylnitrosamine induced rat model of liver fibrosis	50 mg/kg	6 weeks	GSK-3β↓, β-catenin↓, cyclin D1↓, c-myc↓	[35,50]
			CCl4-induced mouse	50 mg/kg	8 weeks	Nrf2↑, NQO1↑, HO-1↑	
			LX-2 cells (culture-activated human hepatic stellate cells)	50 μM	24, 48 h	GFAP↓, Wnt5a/b↓, Wnt3↓, GSK-3β↓, β-catenin↓	
Quercetin	Flavonoid	Fruits and vegetables	Thioacetamide induced	50 mg/kg	4 weeks	shh↓, Ihh↓, Ptch-1↓, Smo↓, Hhip↓, Gli-3↓, TNF-α↓	[51]
Salvianolic acid A		Salvia miltiorrhiza	CCl4-induced	20, 40 mg/kg	6 weeks	NF-κB↓ (in the nucleus), p-NF-κBp65↓, IL-1β↓, IL-6↓, TNF-α↓, TGF-β↓, Cox-2↓, p-JAK1↓, p-STAT3↓	[52]
				5, 15 mg/kg	6 weeks	p-AKT↓, p-mTOR↓, p-p70S6K1↓, caspase 3↓, Bax↓, α-SMA↓, PDGF-β↓, desmin↓, vimentin↓, TGF-β1↓, Bcl-2↑	[53]
Isochlorogenic acid A	Phenolic acid	Coffee beans, honeysuckle, tobacco leaves and kiwi	CCl4-induced	10, 20, 40 mg/kg	8 weeks	(NF-κB) p65↓, IκBα↓, HMGB1↓, TLR4↓, NF-κB↓	[54]
Xanthohumol	Flavonoids	Hop derived	primary human hepatocytes (PHH) and HSC	5 and 10 mM	3 day	collagen type I↓, α-SMA↓, MCP-1↓, IL-8↓	[55]
Naringin	Flavonoids	Grapefruit and oranges	thioacetamide (TAA)-induced	40 mg/kg	6 weeks	ROS↓, p-Akt↓, IL-6↓, caspase-3↓, IL-10↑	[56]
Curcumin/Rutin	Flavonoids	Roots of curcumin spp/Flos Sophorae Immaturus	Hepatic stellate cells			PI3K-Class I↓, Akt↓, p-mTOR↓, TLRs↓, MyD88↓, NF-κB↓, TNF-α↓, IL-1β↓	[57,58]
Procyanidin B2	Flavonoid	Proanthocyanidin	CCl4-induced mouse	50, 100 and 150 mg/kg	4 weeks	VEGF↓, HIF-1α↓, α-SMA↓, Col-1↓, TGF-β1↓, CD31↓, Smo↓, GLI1↓	[59]
			human hepatic stellate cell (HSC) line (LX2 cells)	60, 80 and 100 μM	24 h	VEGF↓, HIF-1α↓, α-SMA↓, Col-1↓, TGF-β1↓	
Hesperetin	Flavanone	Pericarp of citrus	CCl4-induced mouse	25, 50, 100 mg/kg	6 weeks	Ptch1↑, Col1α1↓, α-SMA↓, ALT↓, AST↓	[60]
			HSC-T6 cells	2.50 μM		Ptch1↑, c-Myc↓, CyclinD1↓	
Salvianolic acid B	Phenolic acid	Salvia miltiorrhiza	CCl4-induced mouse	60 mg/kg	6 weeks	ALB↓, ALT↓, AST↓, Shh↓, Ptch1↓, Smo ↓, Gli1↓, TGF-β1↓	[61]
			CCl4	100 mg/kg	8 weeks	PTCH1↑, Smo↓, Gli2↓	
Phillygenin	Phenolic acid	Weeping forsythia capsule	CCl4-induced mouse	10, 20, 40 mg/kg	4 weeks	LPS↓, IL-1 β↓, IL-6↓, TNF- α↓	[62]

## Data Availability

Not applicable.
